# A Treatise on Insanity, Its Seat, Symptoms, Causes and Treatment

**Published:** 1823-03-01

**Authors:** 


### Medico-Chirurgical Review,

AND

### JOURNAL OF MEDICAL SCIENCE.

(Analytical Series.)

"Nec Aranearum textus ideò melior, quia ex se fila fingunt; nec noster vilior, quia ex alienis libamus, ut apes."

Vol. III.] MARCH 1, 1823. [No. 12.

### I.

*De la Folie, &c. Reflections on Insanity, its Seat and Symptoms, Mode of Action of its Causes, its Progress and Termination, the Differences by which it is distinguished from Acute Delirium, the proper Methods of Treatment, with Pathological Researches.* By M. Georget, M.D. &c. Paris, 1820.

Those "written troubles of the brain" in which "words are not thought, nor thought the mind," have been hid in vast lazar-houses of human woe, rather as fitted for eternal concealment and for one hopeless course, than as subjects of medical speculation. Mental diseases, however, are now matters of scientific regard, and we hail this as one of the most fortunate essays at discovery and illustration. The author has observed mental alienation for many years in a vast establishment.

"Continually residing," says he, "in the midst of 1200 patients, I have witnessed, repeatedly, every fact advanced, and I have endeavoured to offer such opinions as are founded upon observation."

This is precisely the probationary method of preparation which most entitles an author to regard, and the sort of experience which is stamped with authenticity.

His work commences with some metaphysical reasonings in support of the anatomical doctrines of Gall and Spurzheim:—but it will be obviously irrelevant for us to hesitate in immediately proceeding to an analysis of the more important and interesting parts, which form the professed topic of the publication.

### Chap. I.—*Symptoms.*

Insanity has its peculiar and inseparable concurrence of phenomena, and certain negative symptoms common to other affections. Want of discrimination between the two, has given birth to voluminous writings on simple varieties of the same phenomena, many of which are mere romances, descriptive of the external deportment of madness, to the exclusion of pathological enquiry or classification. The symptoms are, 1st, local, essential, idiopathic, and cerebral; 2d, general, remote, or sympathetic. The brain is the *immediate seat;* delirium, vigilance, disagreeable sensations in the head, as heat, tension, weight, diminution of sensibility and of muscular contractility, variations of the color and temperature of the skin of the face and head, congestion and inflammatory irritation of the same parts, epitomize general symptoms.

1. *Delirium.*—Expression and intellectual physiognomy are too irregular to merit observation. Those whose faculties are alienated, seldom mistake men for women, but in a stranger they see either a friend or an enemy, in a house a palace; others think they hear voices in the air, to which they reply, &c. and, however false the perception, persuasion to the contrary never convinces; the patient always has a ready subterfuge. Sentiment and the natural affections are generally alienated from the nearest ties, and jealousy, indifference, and dislike, are substituted without external motive. The taste for different pursuits, &c. vary. Symptoms of return to the latter, are *very favourable indications*. The exaltation of any moral or physical passion, greatly aggravates insanity; thence satyriasis, nymphomania, and insane royalty. Vivacious insanity is *most rare*, misanthropical *most abundant*. The powers of comparison are defective, the ideas being scattered, incoherent, and unconnected with the present sensations. Mono-maniacs reason, but always upon some imaginary basis. Thus, the *hallucinè* argues with fancied beings, &c. Mono-maniacs are distinguished by being mad on particular topics only—and reason very sanely on all others, chiefly endeavouring to convince individuals that their health is sound. Retrospective memory is generally alienated, or circumstances are perpetually recalled and perverted. The recollection of events in the period of delirium, is preserved after recovery, with the motives of actions. The majority conceive themselves well, feel indignant at their state, and attribute errors in conduct to their unjust confinement; many perceive and ridicule the want of reason in their companions. Some few acknowledge their malady. This disposition, with gratitude for what has been done for them, are most favorable manifestations. They occasionally commit actions without motive, or are impelled by false ideas of the tendency,—thus the mother kills her child that it may go to Heaven. (Dr. Creighton would remark, that self is rather the object in these actions, and perhaps more correctly,—as frequently instanced by the homicides of the religious insane in this country.) The mental faculties are often exalted by a morbid excitement of the imaginative faculty; this class calculate, invent, &c. but in the former, they are much oftener weakened or obliterated. M. Esquirol attributes intellectual disorders to impaired attention, but this is effect and not cause. Intensity and extent of the symptoms enumerated, vary infinitely in degree from the slightest insanity to the most outrageous—but any of these sufficiently identify the disease,—in whatever degree present. M. Georget adopts M. Pinel's classification, with some slight modifications.—The first is:

*Idiotcy.* Defective development of the intellectual faculties, want of ideas; sensations and affections partially bestowed. "Between the want of intelligence up to an extraordinary development of this function, so many degrees exist, that a scale may easily be graduated, the last step of which would be occupied by the idiot, and the first by the most extraordinary genius."

Idiots may be classed thus: 1st. Those who neglect even the gratification of the animal appetites,—and are prone to perish by self neglect. 2dly. Those that suggest their wants but never seek to gratify them. 3dly. Those that possess the qualities in which the two first species are wanting, and betray some sentiment and intelligence. Individuals of these, like David Galbetlie, sing wild catches, &c. 4thly. The imbecile, a common variety, are capable of going on errands, and performing such offices as require little effort of understanding. These possess all the mental faculties in a degree, and, also, the natural passions,—and propagate if permitted.

II. *Mania.*—Delirium; sensations and ideas confused, incoherent, and following in rapid succession. Excitement and agitation, expressed by disordered motions, cries, singing, menaces, and fury.

> 'Tis a tale told by an idiot,
> Full of strange words and fury.

The description of mania is rather eloquent.

"The maniac seems to live in another world; he has forgotten every event of his existence, and the objects of his affections; if he recalls either to his recollection, it is transiently, and without an apparent intention. The exercise of the intellectual faculties represents a chaos. Schemes are formed inordinately, without motive or end; judgment is erroneous, resolutions are vague, and he is alike careless as to the past or the future." 106.

Mania admits of long intermissions of calmness, and of improved reasoning. Like the decided idiot they disregard personal cleanliness. The *furor* of mania implies a delirious exasperation against some object, real or imaginary, founded on false ideas. Idiots are not susceptible of these paroxysms.

III. *Monomania.* In this species, the patient is insane upon a particular topic, and sane on all other points. It is the most common species: it is characterized by pride and vanity in men, and love and religion in females. The varieties include nostalgia, misanthropy, spleen, fanaticism, and satyriasis. The mania, *without delirium,* of M. Pinel, is a variety characterized by ferocity, or proneness to the destruction of fellow creatures. Caligula, Nero, and Louis XI. were monomaniacs of this class. Mania founded on religious dread of another state of being, melancholy, or *monomanie avec tristesse,* are appropriate species of this genus. Monomania with excitement, consists in expressive apprehension of injury, &c. Melancholy is constituted by chimerical fears or griefs, with depression. Suicide is the effect of transient monomania. All the varieties tend to suicide, and patients exhibit great art in effecting their purpose. The spleen of the English, especially in retirement from active life, favors such propensity.

"Want of moral reflection, which conduces to atheism, materialism, and contempt of existence, favors this tendency."

The author ascribes the spleen of our countrymen to causes altogether false. We know of no such thing as "of millions reduced to mendicity," or "of habitual immorality and debauchery, especially among the higher classes, tending to degrade the soul, impair the frame, and lead to violent terminations of existence." *In no other country, is wealth or morality, or any other advantage pertaining to human nature, whatsoever, so fairly diffused.* That we have a bad climate, some fools, several materialists, and many radicals, is doubtless.

IV. *Stupidity.* All manifestation of the thinking principle is wanting. The mind, in fact, appears to be all but annihilated, indifferent and passive to surrounding objects, with the exterior appearance of perfect repose. Adele Fouchet, 36 years of age, entered La Salpetriere, September 1817, for the fifth time, in the above state, exhibiting general insensibility, answering no questions, and never changing the situation in which she was first placed.

"A seton was put into her neck without any expression of pain. Her malady, at the expiration of three months, terminated in ptyalism and cephalalgia; her understanding was restored to its ordinary activity. She was able to relate her past situation. She thought of nothing; when spoken to, she retained the first word of the sentence, but had no power to reply; she felt no pain when the seton was introduced. This is the sole example which has occurred to me of recovery of stupidity as thus characterized." 117.

Our readers will find in the interesting physiological essay of Dr. Jenner on Artificial Eruptions, a similar fact respecting setons in hysteria, &c. there noticed. If the brain is partially compressed, external irritability is augmented; if fully compressed, obliterated.—*(See Dr. J's Essay.)* The spontaneous cure in this case is evidently attributable to change of determination, a principle well illustrated in Dr. Parry's Elements of Pathology.

V. *Dementation.* Superannuation, imbecility, and decay of the intellect; the reasoning principle defective, forgetfulness of the past, indifference to the future; in fact, " second childishness, mere oblivion." It is a consequence of diseases of the brain, of apoplexy, inflammation, &c. The condition of the brain in superannuation is favourable to the production of paralysis.

*Vigilance.* Idiots being insensible to their own afflictions, sleep most of their time. Vigilance accompanies mania, mono-mania, and stupidity; the latter being considered the acute form of idiotcy. Vigilance may continue from the setting in of the last named maladies to convalescence, especially if there be much excitement. Months, nay years, may elapse without a wink of sleep. Sleep, if obtained, may be partial, disturbed, &c. Its return with diminished delirium, is a certain sign of convalescence; if *alone*, it generally announces termination *par la demence.* If wanting in our otherwise improved state, relapse is indicated.

*Cephalalgia.* Very frequent, especially with females. Intense head-aches probably exist for years, before delirium is provoked by moral causes. In the excited stage the brain is incapable of perceiving its peculiar sufferings.

In convalescence the patient is more sensible of symptoms. They decrease as the former advances. They vary in situation, chiefly occupying the forehead and vertex, but rarely the frontal region (susorbitaire) as in certain gastric affections. They are sometimes more interior, pulsating and increased by motions of the head. When on one side (hemicrania) paralysis is to be apprehended. They are most frequent at night and evening.

*Lesions of Sensibility.* Idiots have little cutaneous sensibility to the stimulus of cold or heat, wounds, &c. Mania, monomania, and stupidity, assimilate at the period of excitement. Patients expose themselves at this time to dangerous degrees of cold unclothed—running the risk of gangrene, &c. Melancholics are particularly regardless of external impressions.—*See Histories by Creighton on Mental Derangement.* Excitement having elapsed, extreme susceptibility often succeeds to insensibility. The eye and even the stomach are then morbidly alive to their specific stimuli.

*Lesions of the Muscular System.* Mental derangement may commence with convulsive attacks. They supervene, however, rarely. Idiots are sometimes epileptic. The most frequent affections of this system are debility or paralysis—the latter happens most frequently to females at the age of 40 or 45, and pronounces incurability. In dementation and idiotcy one half are paralytic.

*Of the Exterior Connexions of the Brain.* The carotid circulation is generally in a state of morbid determination. "At whatever period it occurs, I feel assured of the existence of cephalalgia and vigilance."

The veins, particularly the jugulars, are turgid, especially in rage. The capillaries of the face are injected ; the cheeks morbidly warm ; and the eyes too animated. Physiognomy differs according to the peculiar turn of the perceptions, expressing royalty in the lunatic king, religion in the devotee, &c.

*General Symptoms and Sympathies.—The Alimentary Canal.* Anorexia, or bulimia; the tongue white, yellow, or black, and occasionally very red, as in acute gastro-intestinal inflammations. Pain, however, is rarely such as to excite complaint. Constipation is common ; diarrhœa rare. These disorders are very fugitive, and are in general soon succeeded by healthy animal habits. Fever, increased action of the heart, and palpitations, are almost certain concomitants of incipient derangement. The skin is apt to become more brown and dry, but the natural color returns with convalescence. Menstruation is almost invariably suppressed or irregular. As a summary the brain may be said to be primarily affected. Why are other functions so little disordered ? The phenomenon is exhibited in all maladies exclusively pertaining to the nervous system, as in epilepsy, hysteria, neuralgia, and paralysis. Health is usually at the ordinary standard between accessions. We are not satisfied with this reasoning, and our observation assures us that absence of alimentary and visceral derangement is very rare in hysteria and epilepsy ; and that it very often constitutes a grand link in the chain of diseased action, exciting cause as well as effect, where the disposition exists in either of those diseases. We need only cite the authority of that skilful practitioner Dr. Hamilton, the author of the Treatise on Purgative Medicines.

*Causes of Mental Derangement.* Hereditary disposition has more influence in producing mental derangement than any other malady. Hereditary derangement occurs more frequently among the rich than the poor, and especially among kings and nobles, who are limited in matrimonial choice.

"How many families are in a deplorable state of intellectual degeneracy! The Jews, habituated for ages, from religious prejudice, to intermarriage, few as they are, present similar examples. It is not uncommon to see at the Salpetriere, two sisters, the mother, daughter, and sometimes the grandmother." 150.

"Hereditary derangement is generally announced in good time by whimsicalities of disposition *(travers dans l'esprit)* certain singularities of character, caprice in taste and habits, peculiar and evilly intentioned conduct, little aptitude to the study of the exact sciences, and an immethodical taste for the arts of display and the pleasures of imagination; sometimes indeed delirium appears to be merely an advanced state of already existing intellectual disorder."

M. Georget attributes the hereditary predisposition, in consistency with the first principles of the work, to original conformation of the brain. Parturition, the crisis of female life, (because, forsooth, women then grow ugly, and fret for the loss of admirers,) superannuation, &c. predispose to mental maladies. Impetuous passions; ungovernable imagination; a vicious education, which does not tend to regulate dispositions, the action of which, in excess is dangerous; a limited object of mental speculation; hazardous enterprizes; the revolutions of states, &c.

"Finally, it may be said insanity never occurs without predisposition; for if it were otherwise, the same efficient causes would always produce the same effects, which does not follow. On the contrary, a moral stimulus which excites this malady in one who is predisposed, causes a fever (ataxiquè) in another, an abdominal inflammation in a third, and nothing at all in a fourth person, who is so constituted as to resist its action."

*Efficient or Cerebral Causes.* Blows, falls upon the head, and compression, may leave behind paralysis, feebleness, or obliteration of one or more intellectual faculties, but dementation is the only consequent form of mental derangement. *Apoplectic or acute paralysis,* but not apoplexy, often announces a relapse of insanity, *(delirè,)* or of that variety of dementation which succeeds *la folie aiguë.*

*Moral and Intellectual Causes.* "Those causes which tend to derange the brain by the very exercise of its own functions, are the most frequent, nay, almost the only causes capable of producing mental alienation." 160.

Ninety-five in one hundred owe their malady to moral commotions. It is excited at an age when the mind is most susceptible of strong emotion, and influenced by powerful interests. Infancy and senility, obviously less affected by such circumstances, are almost exempt. As moral causes have been depreciated, physical have been unduly magnified, but it is evident that hereditary predisposition, parturition, and critical periods, merely increase the frequency of moral affections. The secret chagrins of the female mind are often very difficult to discover; jealousy, vanity, clandestine attachments, a lover's perfidies, often form the latent source. The desire of sexual union, increased by reading, and especially by the *spectacles* of France, often excite melancholy in a form which tends to conceal the real cause.

Moral causes are almost as numerous as the acts of the understanding itself; emotions which operate suddenly and forcibly, or slowly and in a sustained manner, surprize, terror, passion, joy, sorrow, and hatred; and, on the contrary, such causes as imperceptibly concentrate and exalt passions or ideas; *e.g.* love crossed, self-love wounded, religious travail, are influential, especially with females of inferior station. Education and affluence create essential differences. The misconduct, debauchery, and brutality of husbands, poverty, &c. affect the lower class; "ambition's honours lost," excess of study, misanthropy, reverse of fortune, almost exclusively affect the higher orders.

"Excess of religious ideas produces different shapes of madness, according to the individual's character. Superstition united with ambition, and the desire of empire, gives birth to intolerant and persecuting fanaticism, to the desire of ruling in God's name, and of making converts. With the subdued spirit, *outrée* religion produces panophobia, fear of divine chastisement, and demonomania. Finally, its singular union with amorous passions, excites ecstatic love of God, the Virgin, or some saint."

M. Georget, of course, holds in view hereditary predisposition as the intermedium between cause and effect. Moral causes act immediately on the brain, whereas their effect on other organs are merely sympathetic—*e. g.* the general expansion in joy, epigastric obstruction in vexation, palpitation of heart in surprise, &c. are absolutely similar to effects excited in the alimentary canal, by any sudden mental irritation.

*Physiological Causes.* Suppressions of secretions, natural drains, menstruation, hæmorrhoids, milk; of artificial discharges, or even diseases, long established.* Irregular menstruation is a most frequent cause; and some interesting cases tend to establish the *dictum* of a moral origin. The same theory is said to apply to all suppressions, natural and artificial. Suppression of the perspiration of the head sometimes causes insanity, similar to the sympathetic derangement consequent to organic lesion.

Alcohol and drunkenness, especially among the English, are exaggerated as causes of insanity, and have led to the hypothesis of its being sympathetic of disease of the alimentary canal.

"Drunkenness affects all organs; the man who is completely intoxicated, has neither sensation, understanding, nor motory power: in mania, on the contrary, intelligence is *false*, but it exists; it is the only function greatly affected; the patient has sensations, walks, talks, and eats. The delirium of inebriation is comparable with the sympathetic delirium of severe diseases, and is as fugitive as the exciting cause itself."

The abuse of alcohol, by enfeebling the general system, and the brain as a part, may end in dementation, *(la demence,)* with paralysis.

*Pathological Causes.* This includes the sympathetic delirium of acute maladies, and of the fatal windings up of chronic diseases, as phthisis, diarrhœa, &c. Females are often sent to La Saltpétrière as *insane*, with low fevers, or abdominal inflammations, attended with *temporary* delirium. Supposed sympathetic deliria are often mere concurrences with other diseases, though simultaneous and independent of each other, as in incipient phthisis, worms, uterine tumours, fractures, &c.

"The epilepsy which accompanies idiotcy is not the cause, but the effect of vicious organization of the encephalon." 171.

The derangement of syphilitic females recently seduced is owing to a moral cause, and not the simultaneous disease. Timid people affected with diseases decidedly mortal, seldom enjoy a calm state of mind. A colicky female fancying her-

* This is certainly inconsistent with what is afterwards said as to the same phenomena being merely progressive effects.—*R.*

self affected with scirrhous pylorus, became deranged from apprehension, and the abdominal affection ceased—one irritation superseding the other; thus insanity becomes a consequence of other maladies.

*Developement, Progress, and Termination of Insanity.* Though moral agents may cause the sudden explosion of insanity, it is more frequently the result of protracted and repeated morbid actions. The latter are preceded by a first period of incubation, obscure in the present state of our knowledge, but meriting observation, since it would tend

"To exhibit the source of various lesions, which are attended with preparatory organic manifestations, which prove that the cerebral function may be deranged long before it is perceptible to society, or even to physicians particularly skilled." 178.

*Second Period of Incubation.* In this period the dawn of derangement is marked by egoistical struggles, inconsistencies, &c. which escape present observation.

"Long before developed insanity, the habitudes, tastes, and passions, undergo a change."

One struggles unsuccessfully to limit desperate speculations; another becomes suddenly highly devout, and endeavours to shake off his fear of damnation, and all in vain.

A young nobleman took a journey the day after his wife's accouchement without any motive; he exhibited inconsistencies of conduct during the journey; six months afterwards insanity burst out. Was not the journey the first act? *Change of character*, endeavour to repress disordered trains of thought, mark this insidious period. The lively become sad; solitary reveries under pretext of study, aversion to locomotion and the presence of others, neglect of their particular profession or pursuit, follow. The period of incubation may last a year or more; corporeal as well as intellectual lesions ensue. Deranged action of the cerebral system, as always happens, is succeeded by disordered functions of other organs. Disturbed sleep, head-aches, morbid determinations, heat of surface, disordered digestion, irregular catamenias, finally terminating in catamenial suppression. The suppression of eruptions, rheumatism, and local gout, are sometimes *effects* of the primary cerebral affection, and occur in the same consecutive order as preceding symptoms.

*Invasion.* Having stated the latent period of insanity, and the primordial connexion of the brain with the development of the disease, we pass from that period in which the patient still continues to be a member of society to that in which he is obviously unfit for its occupations. Melancholy presents the most dubious manifestations; but insulated actions and words escape which denote its presence. The *aliené* now reasons on and defends his eccentricities, and believes the reality of his perceptions; vigilance, cephalalgia, disordered functions, loquacity, laughter, ecstacies, deep anguish, now occur. Disorder of the brain predominates in this stage; the development is not generally sudden, for the *organ appears to struggle against disease as long as possible.*

*Period of Excitation.* Cephalalgia, delirium, and all the corporeal symptoms increased.

*Decline.* The bodily organs becoming habituated to the cerebral malady, recover their healthy actions. Mental derangement remains.

*Terminations.* Recovery or fixation in an incurable chronic state. The former may ensue as a sudden and spontaneous effort of the system, or as an effect of moral agitation, or mechanical concussion. An *aliené stupide* recovered in consequence of a fall against a window but relapsed.* A young woman fell into profound melancholy, in consequence of crossed affection;—her lover's presence, and marriage restored her—but, says the author, (doubting that this was real insanity,)

"Marriage may prevent insanity, but I cannot believe that it will cure it, if once elicited. The patient is then incapable of judging of that which he is told, or of his own actions."

Sudden recoveries seldom hold like the more *gradual.* Convalescence is announced by the gradual, moral, and physical improvement, and in symptoms already expressed—sound sleep is *most favourable; if wanting or disturbed, convalescence is always treacherous.* The irregular bodily motions cease, the colour and smoothness of the skin returns, though pale. The peculiar convulsed and tense features of insanity yield to a calm, though expressive physiognomy. Delirious immodesty in females is succeeded by the very reverse, &c. Expression, however, is often deceptive, the most tranquil appearance may be disturbed by incoherent ideas. The patient becomes more lean in convalescence. M. Georget reasons with great good sense against the doc-

* This happened to a tradesman of Gloucester, sometime idiotic; who, in consequence of a violent push from a stranger, who was indignant at seeing an apparently rational individual playing with boys in the street, jumped up in his sound senses, and returned to his occupation.—*Sir A. Cooper's Lect. Auth. Dr. Cheston.*

trine of *Crisis.* Insanity terminates according to the following law:—

"I have constantly observed that Nature proceeds slowly in the re-establishment of organs; the duration of diseases is relative to the intensity and nature of the organic lesion; the individual constitution and external circumstances favourable or adverse."

The restoration of certain secretions, as of suppressed nasal profluvia in catarrh, &c. are to be viewed as effects and not causes of spontaneous favourable changes. That the favourable change, however, at the eruptive period of the exanthemata, merely indicate periodical coincidences, without any other relation, would be against the general design which Nature always displays, and *which it were useless to imitate*, if not true. As M. Georget's reasonings on this point are not very lucid, we recommend to him Dr. Jenner's luminous observations on this head.

Fifteen or twenty only, out of three hundred cases of insanity, appeared to M. Georget, to be indebted to critical changes. One followed the frequent application of opiate cataplasms, another *grangrenous phlyctenœ* from severe cold, and others *boils or furuncles.*—The truth is, all diseases purely pertaining to the nervous system are very much disposed to yield to supervening affections of another nature, whether natural or artificial.*

The brain which is first affected is also last affected in convalescence. Reproach and false construction on the actions of convalescents should be avoided, and they should also be *removed to a new scene.*

No malady presents relapses or recurrences so frequently as mental derangement. Apoplexies and neuralgiæ observe similar laws. The chronic or incurable state of delirium, mania, or monomania, usually terminates in dementation—if life is protracted to a sufficient period, *i. e.* to abolition or imbecility of the faculties. The class of incurables comprehends idiots, (generally paralytic) the passive, (occasionally reasonable,) and sociable patients. *La demence* is generally preceded or followed by paralysis; is sudden or slow, or supervenes on apparent convalescence. When the disease degenerates into this state, cerebral energy is irrecoverable. The type of insanity is most frequently *continued*, sometimes remittent or intermittent. Accessions are marked by morbid determinations to the head, with the usual symptoms.

---

* In the Pontine marshes intermittents exhibit the truth of this assertion, when they affect patients with nervous diseases, in a remarkable manner.—*R.*

*Prognosis*—Recovery is most frequent from twenty to thirty years of age, rarely after fifty. Mental derangement with paralysis, epilepsy, idiotcy, and *la demence* is incurable. Physical recovery without mental improvement augurs badly.

Patients apparently phthisical, were cured of this disease by the application of the actual cautery to the thorax, but the alienation remained. Maniacal patients, mono-maniacs with excitement, (*exaltation*,) stand a better chance than melancholics or lypemaniacs; but of 1223 females cured, 604 recovered in the first year, 502 the second, 86 in the third, and 41 in the following seven. Spring and Autumn are most favourable; Winter most unfavourable, to recovery. Reports of treatment vary according to the nature of the establishment, some admitting incurable cases, others, curable. At the Salpetrière, the *aliénées incurables*: viz. idiots, epileptics, paralytics, and all above 50 years of age, are separated. Of the other varieties at this establishment, *one half are cured*, according to the average experience of 20 years—a result not exceeded, perhaps, in success elsewhere.

*Diagnosis between Insanity and Acute Delirium.*—These affections are not easily confounded, save in intermediate cases. The latter consists in a derangement of intellect, similar to insanity, but differing entirely in manifestation, causes, and progress. It is the effect either of affections of the brain, of diseases of other organs, or of the action of certain substances on the stomach. Serious diseases of the brain afford either coma or acute delirium. Compression from any cause excites the former, arachnitis, cephalitis, ataxique fever, &c. the latter. Few acute and chronic diseases terminate fatally without delirium. " It would seem that Nature wished, by this means, to veil the approach of the fatal point." Phthisical patients are said rarely to endure delirium at the close; according to our observation, this is a mistake. All organizations do not equally affect the brain in their derangements; inflamed serous membranes, acute inflammations of the alimentary tube, readily excite it. Not so affections of the lungs, nor chronic local maladies; hypochondriasis may supervene on the latter. Acute delirium is, of course, present with the symptoms of that disease which excites it.

" It is always accompanied with excitement and cerebral congestion. When not continued, it usually supervenes, in paroxysms, at evening or night, the circulation, at that time, being most active." P. 233.

" Every function is deranged when any disease is so intense as to cause delirium."

The functions of the skin are suppressed. The mucous tube is covered with secretions, which vary from yellow to black hues. If the malady takes an unfavourable turn, the pharynx or œsophagus becomes paralytic or convulsed. Black and fetid dejections usually occur at this time. The bladder is sometimes paralysed. Delirium is, in general, a dangerous symptom. "It announces the approaching and unhappy end of chronic affections. More than half of those who have it in a continued form, perish." As this applies to delirium symptomatic of organic affections of the brain, the estimate is probably correct. The good effect of repeated small bleedings, almost to exsanguination, we have lately seen in a case of long-continued intermitting delirium, which must have otherwise proved fatal.*

The treatment of symptomatic delirium must, of course, be addressed to the organic lesion, from which it arises. Stimuli, as musk, camphor, &c. increase it. If irritation produces evident congestion of the brain, local bleeding and gentle reduction are indicated. There is a species of delirium, independant of serious lesion of other functions, and probably, a variety of *ataxique* fever, in which, tranquillizing treatment, and opiate *lavemens* produce the best effects—as in delirium tremens.† A table of minute diagnoses between acute delirium and insanity is drawn up.

*Treatment of Insanity.*—Medical enquiry in every form, should be directed to this difficult object. In the affections under consideration nothing is more necessary or arduous. Ignorance of the cause, has led to endless specific and fanciful methods of treatment; thence, organic causes have been attacked by such engines from the arsenal of therapeutics, as *douche* baths, and cold baths, baths of surprise, falls, leaps, rotatory swings, &c.‡ Messrs. Pinel and Esquirol adopted principles of treatment more consistent with the philosophical progress of medical science. These are regulated by know-

---

* A case, probably, of chronic inflammation of the brain, according with cases described by Dr. Abercrombie on that head.

† Mr. Hodges, an ingenious practitioner at Maidstone, and assistant surgeon to the West Kent Militia, has informed us, that a fever, which accords with this description, followed drunkenness in his regiment, after exposure to typhus in a low situation. Several were bled and invariably died,—others were treated with antimonial emetics and opium, and invariably recovered.

‡ A case is related of an epileptic brought to the La Salpêtrière with a stomach eroded and stripped of its mucous coat by a long-continued course of lunar caustic. This is an excellent illustration of the folly, wickedness, and inefficacy of quackery.

ledge of the seat and nature of the disease, the mode of action of its causes, and the individual relation to sex, age, temperament, &c. To ascertain the morbid state of an organ, too exclusive importance is not to be attached to derangements of its action; these announce disease, but do not indicate what? Many cerebral lesions are attended with delirium; pulmonary, with dyspnœa, &c. Unexpected phenomena in the diseased organ, as pain, change of texture and volume, disorders in the neighbourhood, afford guides to the desired discrimination. M. Georget vaguely says, in treating insanity,

"We are to consult the absence or presence of excitement or asthenia, and not the species of delirium."

"The treatment of a malady, produced by any adventitious cause, requires no modification, thus, a pleurisy from suppressed menstruation, or suppressed transpiration, claims the same curative processes as simple pleurisy. It is vain to attempt to re-establish menstruation or transpiration; both would observe their usual periods. The removal even of a fixed (persistante) cause, does not always remove the disease; once altered to a certain extent, organization does not return to its natural condition, but with a determinate progress. A foreign body applied to the conjunctiva may cause ophthalmia, but its removal will not cure it."* 251.

This is very fashionable reasoning, especially among those who cannot, or will not, think or see for themselves. Doubtless it is true to a certain extent; but acute inflammation of an internal organ often ceases when brought back to the extremities, and inflammation following suppressed menstruation, often ceases when the hip-bath, aloetic clysters, hellebore, and leeching the pudenda, restore the determination. We saw membranous ophthalmia lately cease immediately after removal of a mechanical irritant, which had been treated a fortnight in vain. To a certain extent, such reasoning is true, but it savors more of logical squeamishness, than matter of fact, and ought not to supersede the more rational indications, the success of which, when acted on, is verified by infinite experience.

The treatment of mental derangement is both moral and physical, but our ignorance of the relation between these two states of the brain, viz. intellectual disorder and cerebral alteration, renders the effects of remedies not only uncertain, but makes it often doubtful, whether that which is beneficial in one point of view, is not injurious in another. Moral

---

* Yet, in page 311, M. Georget says, "if you remove a foreign body from the conjunctiva in time, you *will prevent ophthalmia, and the eye will suffer nothing.*"

treatment is more determinately useful than physical, which must be secondary and addressed to symptoms.

*Direct Cerebral Treatment, moral and intellectual.*—Cures effected by accidental agents do not admit of imitation; a fool is not to be thrown out of a window, or the house of a paralytic to be set on fire, because one recovered his senses, and the other the use of his legs, by such accidents. Philosophical treatment consists in lessening and extinguishing the causes which impelled the development of the disease, and which tend to keep it up, and renew it. Amatory and religious insanity are especially perverse. One requires great strength of soul, the other never gets well without an almost complete change to a state of religious indifference. That from jealousy is similarly obstinate. The indications are, to separate the patient from all objects which operate as causes or motives; to restrain formidable actions; to rectify errors of sense; to fix attention upon a limited number of objects; to encourage reflection on personal conduct and thoughts; and prevent mental aberration;—to excite idiots to think, melancholics to firmness and hope; finally, to restore their habitual thoughts and particular attachments. These indications demand two methods of treatment, passive, or isolation; active, or medical education. The former is almost indispensable in all cases, to facilitate oblivion of past impressions and causes, and excite new. The confidence of possessing authority, is superseded by isolation; and deference exacted by it to guardians.* Isolation by travel is but suited to the splenetic. Particular establishments, unless regulated with attention to reciprocity of condition, do harm. Nothing avails so much as the society of those whose sufferings correspond. The special establishments of France are maintained at the cost of government, especially those which receive the indigent. The Parisian, are the Bicetre for males, La Salpétrière for females, and the Clarenton for both. The departments are

---

* The abuses of asylums are prevented in France by legislative control. A gentleman in full possession of his intellects, in Lincolnshire, was put into a house famed for "des manœuvres, aussi viles que odieuses." He wrote to a friend who knew the nature of these intrigues, and who, being well aware that, whether mad or not, he never could get out during life, immediately wrote to the keeper, that the affairs of the patient would never enable his friends to discharge the costs. The consequence was, that the patient immediately got free! The house, we were informed, exists in the capital of a neighbouring kingdom, which exhibits men degraded to bestiality, and treated with as little consideration. This too is daily visited by hospital surgeons, men of education and liberal sentiments, who, for reasons best known to themselves, see and say nothing!!!

deficient, and deranged persons are thrown *pêle-mêle*, into the worst parts of hospitals. La Salpétrière contains 1200 persons, and is separated into two sections; the one destined for idiots and *alienées en demence;* the other for the maniacal, mono-maniacal, and the *alienées stupides*, incurable or under treatment. The latter is subdivided into large dormitories, for the classes under treatment and convalescents, and separate beds for the refractory. Gardens, baths, promenades, &c. are attached. It is suggested that these establishments should be built on the ground-floor, and have no large dormitories, because one violent patient disturbs all the rest; that the incurables should be entirely distinct, &c.* La Salpétrière is directed by a physician, who has notice of all alterations and punishments, receives parental expostulations, &c. There is also a chief inspector, of mild and amiable habits, sub-inspectors, and female servants. The second class protect the timid from the violent, and repress outrageous conduct: the former is the centre of general confidence and authority. Mildness, persuasion, and strict fidelity in promises, &c. is advised. The turbulent are secured by suddenly enveloping the head; they yield immediately when they cannot see where to strike. Change of rooms, the grated court, strait waistcoat, bath, and seclusion, are the only corrective means had recourse to. Men most readily submit to females and *vice versa.* "The latter, seldom entertaining a good opinion of their own sex, usually ascribe the worst faults to their governesses, with whom they are almost invariably on ill terms."

*Medical Education, or Treatment.* This requires profound intimacy with human dispositions, motives, and affections. It applies to that period of the disease when excitement and general or cerebral irritation is abated, when the ideas are less fixed and tenacious. Patients should never be flattered in the false constructions which they put on their own situation; by such a course lover, devotee, king, &c. are confirmed in their deranged personifications. The exercise of such ideas is to be prevented; yet, on the other hand, vehement contradiction merely exasperates to no purpose; but the best of all manifestations, *viz.* doubt of the correctness of their own perceptions, points at the period for persuasion and conviction of error. (Who does not recollect the well-timed arguments of Imlac to the astronomer in Rasselas?) The excitement of new turns of thought, the rousing of inert faculties form a

* See Art. Hospital Aliené de M. Esquirol.—*Dict. des Sciences Med.*

third principle of medical education. For instance, endeavour to convince a king that he is without power, with the hope of his reflecting that he may have been in error. Take *hallucinées* to the situations whence the object of the hallucination proceeds, *e. g*. fancied enemies, voices, &c. search and assure them of the non-reality. Awaken the passions of *aliénées stupides* by reproaching them with indifference to parents, &c. The physician is recognized by the worst patients; and readily obeyed when any other influence is defied. He possesses great power in exciting their confidence: and may make excellent impressions by oracular efforts; *e.* g. by relating to them their past conduct, telling them of their designs, as self-murder, destruction of children, hatred of husbands, &c. Cures are sometimes thus effected. The physician ought to pass some time in the midst of these persons; study the motives of their actions, the varieties of character, and compel them to perform his injunctions and their own promises. Nothing accelerates recovery more than the mutual association of convalescents; for this purpose gardens and refectories are especially necessary. Mechanical employments are of the greatest use, except with individuals whose rank interdicts such pursuits. Bodily amusements suit the latter class. Travel, where fortune permits, is an excellent method of relaxing the mind. Family interviews may be granted when the patient is so far recovered as to demand them. Approach should always be announced. At this period very digestible aliment in small quantities should be used. Agitations are to be avoided.

"Happily in France these unfortunate persons are not *exhibited like curious beasts*."* 294.

*Cerebral Treatment*. Excessive bleeding, purging, and the use of pretended specifics has constituted the empirical treatment of insanity. M. Pinel and Esquirol reduced the treatment, first in France, and almost the first in Europe, to physiological principles, but without the wished-for success, from the nature of the disease. Asphyxia produced by hanging, drowning, or blows, the practice of our quacks, is justly condemned. As is usual, these feats are said never to fail; one cures every patient with emetics,† another with vinegar, &c.

---

* This does not occur so unreservedly in England, since the exposure of the infamies of the old Bethlem Hospital.

† Sir Astley Cooper has told us that the use of tart. emetic *internally* in a large Establishment merely suspended insanity during its action on the stomach.—*R.*

This part of the subject is involved in great difficulties; we are ignorant of the *quo modo* of the functions, moral and physical, of the organization affected. Treatment is consequently indirect and aimed at a particular lesion wholly dissimilar to any other morbid actions. Indications are therefore guided by symptomatic effects, purely physical, as morbid determinations, &c. Much is promised hereafter from the portfolio of M. Esquirol, and the Dictionnaire des Sciences Med. The author does not profess to have advanced science in this respect.

"Diffident of the experience of others, I shall limit myself to that which I have seen. I shall offer no miracles; they are not made for rational people."

Individual remedies are all of very limited use. At *la Salpétrière* M. Fouquier's nux vomica *has failed to cure in any case of paralysis!* The use of violent remedies is less successful than attention to the prevention of untoward circumstances, and conducting the disease, by well-timed interferences, to a fortunate issue. Attention to the due regulation of the mental and bodily functions forms the grand outline. Any active symptoms which concur or supervene, as cerebral congestions or organic inflammations, will demand the most active practice.

*Hygiene.* The general laws apply to this as to other diseases. Diet and exercise should be voluntary, thereby avoiding irritation. Nudity, if desired by the patient, may be granted—with care to decency, and in winter to the protection of the extremities. Extreme cleanliness. Beds of oaten straw are used for the unmanageable. Narcotics have a variable effect, but are generally less active than with others. Emetics, epispastics, and purgatives, produce the ordinary influence, except that their local action does not seem to exert the same general influence on the brain, and excites less sensation. In the period of incubation, removal of moral causes, as reparation of injury, or re-union of lovers, avails infinitely more than tormenting the organic system with stimuli addressed to functions impeded or suppressed merely as effects.

*Period of Excitement.* Sequestration, restraint, diminution of irritation spasm and convulsive actions. Privation of light and heat, cropping the hair, and exposure of the head, (if wished,) acidulous beverages, with fruit, but no wine. Tepid bath daily, if strength permits, staying in as long as possible, *from half an hour to two or more.* If an apoplectic tendency obtains, it is interdicted.

"The use of baths tends especially to diminish excitement, to calm the nervous system, to dissipate tension, agitation, and encreased muscular action; to relax and refresh the dry skin of melancholics. It is of use, and often essential in point of cleanliness." 316.

This stage does not require violent treatment. Blood-letting has been much abused in it, and has produced the worst effects—confirming the derangment of mind, and dangerously enfeebling the system, except in supervening acute affections. In congestions, local determinations, and inflammations of structures, *e. g.* serous membranes, &c. local blood-letting is advised. Pervigilium, which is increased by opiates, requires blood-letting. Dyspepsia, suppressed menstruation, will only be abated by diminishing cerebral irritation, and by the successful progress of the general plan. The author's objections to external irritants and cold affusion, are absurd. "Nine hours' cataract with the shower bath" is certainly extravagant enough. Baths of surprise and empirical means need no observation. General plethora, common at pubertal periods, require repeated small blood-lettings.* Bleeding from the foot, and cupping the thighs, after suppressed catamenia. Baths in plethora are manifestly improper—disposing to internal congestions and apoplexy. Debility with the usual symptoms, and delirium or loquacity, may follow refusal of nourishment, masturbation, or subterranean confinement, or improper depleting treatment. Masturbation in both sexes is generally excessive, and requires restraint and cold applications to the genital system. If the parotids are pressed, pain compels them to open their mouths, and the introduction of a tube will ensure deglutition. Active cerebral congestion, premonitory of apoplexy, and common to all varieties, demands derivative treatment, by new irritations, nauseating remedies, irritative or mustard pediluvia, (cepheluvia *being used simultaneously*,) and foot-bleeding. Small blisters to the arms or thighs, and neck (occasionally.) Sub-inflammation of the brain, denoted by encephalic tension, head-ache, and the phrenitic physiognomy with vibrating carotids, requires the same treatment.

---

* The author has well described pubertal plethora, and likewise that at the turn of life; fulness with a sense of sinking and lassitude is present, and oft mistaken for general debility, and most injuriously treated as such. We have found no remedy equal to the sulph. ferri (one grain dose) with the aloetic and sagapenum pill of Dr. G. Fordyce, in these cases. It produces free purging, occasional alvine hæmorrhage, and thus lessens congestions of the spleen, and abdominal viscera.

*For the stupor, insensibility, and cerebral inactivity of aliénées stupides;* setons, moxa, &c. to the neck, *frequently repeated and kept up;* they cause a general shock, but require time for constitutional influence. These, and repeated vomits, rouse the animal energies in the "most desperate cases."

*Nervous Irritability.* This is accompanied by leanness, despondency, morbid susceptibility, mental or bodily impressions, despair, constipation, fidgets, dyspepsia, and watchfulness. The treatment which is here indicated, is purging, tranquillizing, obviating local actions, diminishing cerebral irritation. But, above all, the hellebore and "Naviget Anticyram" of Horace, viz. mental and bodily transitions by travel.* Jalap, hellebore, aloes, and colocynth, (*daily* if not too effective,) produce serous evacuations abundantly, and relieve the cerebral action. At night the system should be calmed by conium, &c. Orange flower *ptisans* excellent in great quantity. Irritants to the skin and baths improper. In cases which are entirely hopeless under the foregoing modes of treatment, the empirical methods may be had recourse to, in as far as they do not endanger life.

*Tendency to la demence.* This state combined *with* paralysis (acute or chronic) is incurable; *without it*—very doubtful. As it implies collapse of the mental powers, tepid baths, tonics, stimulants, derivatives, and counter-irritants, are indicated.

*Derangement consequent to Parturition*—is generally more curable than any other, especially if taken early after the event.

*Treatment.* Excite the secretions of the intestines and skin; give daily *lavemens* of milk and sugar, which cause abundant stools without irritation—tepid bath. *The general Treatment of Excitement.*—Renewed blisters to the arms. Breasts hard, painful, and slow in advancing to resolution, require friction and ammoniacal liniments: opiate cerate for dressing. Under this treatment hysteritis or peritonitis rarely follows. Those who become insane after every parturition should abstain to prevent the effect.

---

* A gentleman at Bath, in whom retrocedent rheumatism, torpid liver, and melancholy of this description concurred, recovered by removal to Cheltenham, where his rheumatism was restored—under desperate despondency. In this state he was *forced from Bath* by our admonitions, in direct innovation on his local advice, which in watering-places seldom tends to prompt removal, however necessary, from feelings of jealousy towards other rival situations.—*R.*

*Periodical Derangement.* Rarely curable. Tonics sometimes prevent the accession. Syncope, convulsions, fever, vomiting, are generally fugitive, and merely require tranquillizing treatment.—*Convalescence.* Atony is usual at this period, with œdema; embarrassment of speech, itching of parts, and painful digestion, often forerun paralysis, as, also, much sleep. Some who recover become phthisical.—*Vigilance*, also, occurs in convalescence. Tepid baths, active exercise, orange-flower water, blisters to the arms.—*Plethora* is characterized by palpitations, weight in the head, disturbed sleep, &c. Low diet, active exercise, *cautious* blood-letting. —*Cephalalgia.* Very general in convalescence. Tranquillity mostly suffices. External head-aches, if obstinate, require pediluvia and leeches to the part affected. If internal, and indicating congestion, active constitutional treatment. Suppressed menstruation, with such symptoms, as cephalalgia and plethora, requires emenagogues, mustard pediluvia, foot-bleeding, hip baths or fomentations, leeches to the pudenda. —*Relapses.* To prevent all the preceding symptoms, remove causes, and regulate the functions. Marriage has been proposed for love-madness. It entails three evils, its own chagrins, the hazards of *accouchement*, and of hereditary transmission. *Guard the brain especially*, and, also, the regular action of the uterus and bowels. Baths, partial and general, crural and pudendal leeching, purges, and setons in the arms.

*Pathological Researches.*—Our analysis of this section must, necessarily, be very brief, especially as the author's opinions entirely refer the origin of mental alienation to organic derangement, not sensible or demonstrable. For his speculations on this point, we, therefore, refer the reader to the work, briefly noticing such pathological particulars as are interesting to all.

The infrequency of manifest organic affections of the brain, and its occurrence in those not affected, leads the author to deem such phenomena as effects, or, at least, merely the causes of such secondary nervous affections, as concur with derangement.

Though mental derangement is not in itself mortal, it necessarily involves exposure to the causes which abridge life. Out of 100 received in the Salpêtrière—

| | | | | |
|---|---|---|---|---|
| 25 die | 1st year. | | Out of 95, | age. |
| 20 | 2d | 20 | die at | 20 to 30 years. |
| 18 | 3d | 31 | | 30 to 40 |
| 14 | 4th | 25 | | 40 to 50 |
| 14 | 5th to 10th | 7 | | 50 to 60 |
| 7 | 10th to 15th | 9 | | 60 to 70 |
| 2 | at the end of 20 years. | 3 | | past 80 |

Acute maladies very rarely supervene compared with chronic. Diagnosis is very difficult, sensibility being diminished, symptoms may not be well marked; some feign symptoms and diseases; morbid alterations of functions, debility, want of appetite, and dejection, afford the best indications of chronic maladies. "Phthisis destroys more than half the patients admitted into the Salpêtrière. It is *never acute*, and often so *latent* that it is not discovered till *examination after death*." Not the least mark of pulmonary irritation exists, the patient neither coughs, expectorates, nor complains; he attenuates, loses strength, and dies with constipation or diarrhœa; the progress is very slow. All who die in bed, die with the last symptom. The first (constipation) is the effect of atony, and is often wonderfully protracted. It is often incurable, the intestines, becoming enormously distended and paralyzed. Mechanical efforts alone avail.

*Crania of Idiots.*—Almost always mal-conformed. Volume either preternaturally small or large. Out of 100 crania of *aliénées*, 50 may be natural, and the rest irregular, thickened, &c. These changes are probably effects of cerebral development, rickets, &c. In the encephalon, the rachidian column, and meninges, except in aged *démences*, and in paralytics, *no organic alterations at all are generally observed*, unless fevers, or other acute diseases, supervene.

*Dura Mater.*—Occasionally thickened and ossified. *Tunica Arachnoides and Pia Mater.*—Chronic inflammation, or serous effusion. *Cerebrum*, sometimes hard, sometimes soft. *Ventricles.* Adhesions, especially at the prolongation of the cornu ammonis, serous effusion, especially in idiots. *Plexus Choroides*—Bloodless. Full of hydatids.—The *lobes* changed in color, and putridly soft, especially in paralytics. Partial atrophy of the substance of the brain, is almost peculiar to paralytic idiots,* the portion affected being reduced to one third of natural bulk, and the centre hard or cartilaginous. Erosion. Cancerous-like tumors.—*Cerebellum* soft.—*Spinal Column.* Rarely affected.—*Pleura and Lungs.* Adhesions and tubercles frequent.—*Intestinal Canal.* Mucous coat almost always more injected than naturally.—*Colon.* Transverse arch occasionally oblique or perpendicular.—*Liver.* Preternaturally large or small—sometimes congested, tuberculous, scirrhous, hydatidous.—*Gall Bladder.* Distended with bile; gall-stones.—*Spleen.* Enlarged, soft.—*Uterus, Ovaries.* Tuberculated, enlarged. The author sums up:—

* "This, which is congenital, is probably the immediate cause of defective understanding, as well as of paralysis."

"All the morbid changes observed, are consecutive to the development of mental derangement, except those in the brains of idiots, which are primary, and connected with the paralytic state."

Chronic cerebral irritation and paralysis, may be effects of such changes. The changes of the thoracic and abdominal organs arise out of accidental circumstances. Finally, the majority of cases exhibit no cerebral changes whatsoever. This chapter, also, contains some interesting enquiries into the extent to which dissection will carry us in the investigation of diseases, and its fallacies.

This book is pretty nearly the best on the subject, not because it abounds in novelty, but, because it consists of dense and genuine experience, and of extensive pathological evidence, viewed and combined with sound physiological judgment, which has enabled the author to develope mental diseases, simply and lucidly, from their dawn to their downgoing. We have in it, a final arbitration of many difficult points. Almost every page has been duly analysed, and the most valuable parts are extracted, and presented to the reader in aid of his practical conduct, whenever these unfortunate maladies form the object of private care.

